# Supplementary material for: The last stretch: Barriers to and facilitators of full immunization among children in Nepal’s Makwanpur District, results from a qualitative study
Source: PLoS One. 2022 Jan 21;17(1):e0261905. doi: 10.1371/journal.pone.0261905 (PMC8782481; doi:10.1371/journal.pone.0261905)
Supplement: S1 File — (DOCX) [file pone.0261905.s001.docx]

Cover Page (Interviewer’s Responses)

Rejoice Architecture Focus Group Discussion Guide for Mothers

Interviewer’s name: _____________________

Interview number: _________

Palika: ____________________

Ward Number: _________

Village: _______________

Health facility name: ____________

Respondent ID: __________

Thank you so much for taking time out of your day to participate in this discussion.

Does anyone have any questions about the research or your participation before we begin?

**Warm-Up Questions**

I’m new to (village name). Can you tell me a little bit about it? For example, what kind of crops do people grow here? What are the places to visit around? What do people like to do for fun?

**Environment**

1. In your community, think about some places that you like and enjoy.

(Probe in case of no response: it could be school, mother’s group meetings, local market or haat bazaar, community events, health facility etc)

- Can you name those places? What makes you enjoy being in those places? *(Ask about each place one by one after the initial response and continue to probe. “Any other place?”)*
- What about the places that you do not like to visit? What makes you not enjoy those places?

**Health Facility Description**

Facilitators: Now, we would like you all to imagine a model health facility and its environment. Remember, we want you to imagine an ideal health facility that you will find encouraging and welcoming. We request you to focus on details that you want both inside and outside the health facility. Also, think about what sort of behaviors you would like to see and include everything that you consider essential to making this an ideal health facility. It is a group discussion, so everyone can provide their inputs on how a model health post should look like. We want to understand everyone’s view on their idea health facility.

1. Now, when you are describing the model health facility , please do keep these things in your mind.
   - What are the aspects that should be which will make you feel welcomed?

- What are the aspects that makes you want to spend time in this health facility?
- What are the aspects that will encourages people to socialize?
- What are the aspects that makes this health facility better than other health facilities?

1. Now, how does your imagination of model health facility compare to your current health facility? Would you like to see changes or improvements to your health facility in those aspects?

- If these changes were made to your health facility, what would change in terms of your health behaviors? Would you be more likely to visit?
- What about your community’s health behaviors?
- Would more children in your community get immunized?

**Facilitators and Barriers**

1. Now I would like you to think about most families in (village name). Who in the family typically takes the children to the health facility when they are ill? Where would they go?
   - PROBE: Government facility? Private facility?
2. What about for immunizations? Who would take the children? Where would they go to receive vaccines?
   - PROBE: Would they visit a health facility or a standalone immunization site?
3. Do most mothers find it difficult to get their child vaccinated?
   - (if yes) What makes it difficult? PROBE: Getting to the facility? Waiting for long periods of time?
   - (if no) Why not? Why is it not difficult?
4. Do you think taking time to get to the health facility and complete the immunization appointment affects most mothers’ personal schedule and responsibilities? How so?
   - Do you think mothers in (village) would be more willing to get their children vaccinated if they could do some of their home chores at the vaccination site? If so, what might those be?
5. We have learned that some women vaccinate their children all the way through 15 months, whereas others stop before all the vaccinations are done. Why do you think this is so? Why do you think some women stop vaccinating their children?
6. Can you tell us about the groups in your community that women are members of? What are those groups? Are there women only groups? If Yes, which are those groups?

- In a typical group of your community, what do women discuss about?
- Do they discuss about health facilities and services including immunization? If yes, what they discuss about? Any examples of previous discussions?
- How do you consider the credibility of information or suggestions discussed in these groups?

**Norms**

1. Do mothers in (village) have access to any information about vaccines? Where could they go for it? Who could they ask?
   - PROBE: Family members? Others who are not family members?
   - Do women receive information about vaccines at the health facility? During prenatal visits? After childbirth? At other immunization visits?
2. Continue to think about most women who live in this community. In your opinion, how many of them vaccinate their children?

- (If some do not vaccinate their child) What could be the reasons some people in your community do not vaccinate their children? PROBE: Time and availability? Beliefs about vaccines? Health workers’ behaviors? Cost?

1. What would the average mother in (village) say about immunization? Would she want her child to be immunized?
   - PROBE: Why would she say this? Why would she want/not want her child to be immunized?

**Caregiver-Provider Communication**

1. What do you think most women experience when they visit with their child’s doctor? What conversations do you think they are having, if any?
   - PROBE: What types of questions did you think they ask, if any? How do you think the providers respond?

- PROBE: Do you think most women receive as much information from the doctor as they want?

1. How comfortable would you say the average woman in your community is with discussing their child’s health concerns with the doctor? Why?
2. What do you think providers could do to make the women in this community feel more comfortable talking with them?

**Health Facility Atmosphere**

Now I want to ask you a few questions about the health facility you visit most often.

1. What emotions do you think the average woman experiences when she gets there? (pleased, uncomfortable, disgusted)
2. How would the women in this community describe the atmosphere at this clinic? Would they describe it as pleasant?

- PROBE: How would they describe the cleanliness of the clinic? The safety? The amount of space?
- What would make the clinic feel more welcoming to the women in your community?

1. Lastly, is there something that could be done that would encourage the mothers in (village) to vaccinate their children?
   - PROBE: Anything else?

Thank you for answering all of my questions and participating in this discussion. Is there anything else anyone would like to add? Do you all have any questions for me?
