# Supplementary material for: The last stretch: Barriers to and facilitators of full immunization among children in Nepal’s Makwanpur District, results from a qualitative study
Source: PLoS One. 2022 Jan 21;17(1):e0261905. doi: 10.1371/journal.pone.0261905 (PMC8782481; doi:10.1371/journal.pone.0261905)
Supplement: S3 File — (DOCX) [file pone.0261905.s003.docx]

Cover Page (Interviewer’s Responses)

Rejoice Architecture Interview Guide

**Health Facility In-Charge or Responsible Health Officer**

Interviewer’s name: _____________________

Interview number: _________

Palika: ____________________

Ward Number: _________

Village:

Health Facility Name:

Respondent ID: __________

Thank you so much for taking time out of your day to participate in this interview.

Do you have any questions about the research and your participation before we begin?

**Warm-Up Questions**

1. Can you tell me about yourself? How long have you been working in this facility? Where were you working before arriving in this facility?
2. In this health facility, what are your key roles and responsibilities?

- PROBE: What are your responsibilities with respect to immunization, if any? Can you explain a bit?

**Immunization status**

1. Are you aware about the immunization status of the catchment area of this health facility? If yes, can you tell me about it? How many of the children in the catchment area would you say are immunized according to national standards?

**Drivers & Barriers**

1. Can you tell me what activities/efforts/initiations that the health facility and its team have made to improve vaccine coverage, if any?

- How effective have these activities been?
- What has worked? What might be the reasons?
- What has not worked? What might be the reasons?

1. Are there any specific groups in the community with lower coverage?
   - If Yes, which are these groups?
   - PROBE: Do they know about vaccines? What are their attitudes toward vaccines? Can they get to vaccination sites? Are vaccines too expensive? What else might be causing these groups to have low coverage?
   - What efforts have you and the health facility made to improve these groups’ coverage, if any?
     1. How effective have these activities been?
     2. What has worked? What might be the reasons?
     3. What has not worked? What might be the reasons?
2. We have learned that some women vaccinate their children all the way through 15 months, whereas others stop before all the vaccinations are done. Why do you think this is so?
   - PROBE: Why do you think some women stop vaccinating their children?
   - What could we do to ensure women come to their child’s next immunization visits? Do you have any experience from similar efforts?

**Norms**

1. Please think about most women who live in this community. In your opinion, what do most women think about vaccines?
   - What is driving these perceptions?
   - What efforts have the health facility made to counter negative perceptions toward vaccines in the community, if any?
     1. How effective have these activities been?
     2. What has worked? What might be the reasons?
     3. What has not worked? What might be the reasons?

**Caregiver-Provider Communication**

1. Can you share with me how the immunization process is organized? Who often brings children to the health facility for immunization? Mothers? Fathers? Someone else?
2. Now, consider your average immunization appointment.
   - What are the routine activities, points of communication, and messages?
   - PROBE: How much information about vaccines is shared with the caregivers? What types of information?
   - On average, how much time do providers at this facility spend with a child during an immunization visit? What determines the amount of time?
     1. What do you think about this amount of time? Is it enough time?
     2. PROBE: why?
     3. PROBE: How does the amount of time spent with the child affect the overall immunization process?

**Health facility Atmosphere**

1. Can you tell me a bit about this health facility? For example, is typically crowded? Would you describe it as clean? How well does it function?
   - In your opinion, what could be done to make the health facilities more friendly toward people?
2. Can you tell me how you feel when you reach the health facility? (Pleasant, Energized, Unpleasant, Tired, Disgusting)

- What makes you feel that way?
- How does this affect the way you work and deal with the clients, if at all? Can you explain?

1. What do you think about the environment of the health facility?

- Is it comfortable and welcoming enough for women who visit for immunization?
- PROBE:
  - 1. External environment (building, garden, space)
    2. Physical facilities
    3. Sanitation and cleanliness
    4. Safety
    5. Materials and equipment
- In your opinion, how does the health facility’s atmosphere affect the experiences of the people seeking services from this health facility?
  - 1. PROBE: Demand for services? Quality of services?
    2. How does it affect immunization, its uptake and continuation?
- What efforts has the health facility and its team made over the years to improve the environment, if any?
  - 1. How effective were these efforts?
    2. What has worked? What might be the reasons?
    3. What has not worked? What might be the reasons?

**Work Load**

1. What is a typical day at work like? How many patients do you see? How many hours do you work? What is the pace of the day like?

- What is your relationship with your co-workers like? What do you like and dislike about it, if anything?
- What is your relationship with your patients/caregivers like? What do you like and dislike about it, if anything?

1. How do you typically feel at the end of a day of work? (PROBE: Do you feel proud, tired, stressed?)
   - How satisfied are you with the way the clinic is run? What can be done to help it run more smoothly?
2. How satisfied are you with the quality of resources available at the clinic? What about the amount of resources?
3. Can you tell me about the health facility and Palika collaboration? How would you describe that relationship?

- Can you tell me if there have been any changes in the ways Palika and the health facility have collaborated in the past few years?
- Any changes in budget compared to earlier? If yes, can you elaborate?

**Intervention Feasibility**

1. We are planning on adjusting the clinic environment and facilitating provider-caregiver interactions in our research. We may paint walls, provide seating, and plant trees to make the clinic more visually appealing. We may also implement a new appointment system and encourage certain ways of speaking with caregivers to improve efficiency and communication. What do you think about these types of changes? What do you think caregivers will say to that?

*If the respondent finds the intervention problematic*

- Why do you think it may be a problem? Is there any way that we can make it more acceptable to you and other providers? To caregivers?

We have come to an end of this discussion. Do you have any questions for me?

Thank you very much for your time and patience.
