# Supplementary material for: The last stretch: Barriers to and facilitators of full immunization among children in Nepal’s Makwanpur District, results from a qualitative study
Source: PLoS One. 2022 Jan 21;17(1):e0261905. doi: 10.1371/journal.pone.0261905 (PMC8782481; doi:10.1371/journal.pone.0261905)
Supplement: S4 File — (DOCX) [file pone.0261905.s004.docx]

Cover Page (Interviewer’s Responses)

Rejoice Architecture Interview Guide

**Palika Representative**

Interviewer’s name: _____________________

Interview number: _________

Palika: ____________________

Ward Number: _________

Village:

Respondent ID: __________

Hello, and thank you for speaking with me today. We requested you to speak with us since you are a Palika representative, and Palika is in-charge of the health facility management committee.

Thank you so much for taking time out of your day to participate in this interview.

Do you have any questions about the research and your participation before we begin?

**Warm-Up Questions**

1. Can you tell me about yourself? How long have you been working with the Palika? What are your primary roles and responsibilities?
2. What are your key roles and responsibilities in the health facility?

- PROBE: What are your responsibilities with respect to immunization, if any? Can you explain a bit?

**Immunization status**

1. Are you aware about the immunization status of the Palika? If yes, can you tell me about it? How many of the children in the catchment area would you say are immunized according to national standards?
2. I have noticed that some health facilities are clean, well-managed, and very efficient, whereas others are not so well run. Can you tell me about the health facilities in this area?
   - PROBE: For example, tell me a bit about whether they are crowded or not, whether they are clean or not, and how they function, overall.
   - In your opinion, what could be done to make the health facilities more friendly toward people?

**Drivers & Barriers**

1. Can you tell me what activities/efforts/initiations that the health facility or Palika team have made to improve vaccine coverage, if any?

- How effective have these activities been?
- What has worked? What might be the reasons?
- What has not worked? What might be the reasons?

1. Are there any specific groups in the community with lower coverage?
   - If Yes, which are these groups?
   - Why do they have low coverage levels?
   - PROBE: Do they know about vaccines? What are their attitudes toward vaccines? Can they get to vaccination sites? Are vaccines too expensive? What else might be causing these groups to have low coverage?
   - What efforts have you and the health facility made to improve these groups’ coverage, if any?
     1. How effective have these activities been?
     2. What has worked? What might be the reasons?
     3. What has not worked? What might be the reasons?
2. We have learned that some women vaccinate their children all the way through 15 months, whereas others stop before all the vaccinations are done. Why do you think this is so?
   - PROBE: Why do you think some women stop vaccinating their children?
   - What could we do to ensure women come to their child’s next immunization visits? Do you have any experience from similar efforts?

**Norms**

1. Please think about most women who live in this community. In your opinion, what do most women think about vaccines?
   - What is driving these perceptions?
   - What efforts have the Palika and the health facility made to counter negative perceptions toward vaccines in the community, if any?
     1. How effective have these activities been?
     2. What has worked? What might be the reasons?
     3. What has not worked? What might be the reasons?

**Health facility Atmosphere**

1. Can you tell me how you feel when you reach the health facility? (Pleasant, Energized, Unpleasant, Tired, Disgusting)

- What makes you feel that way?
- Do you think the facility environment affects the way health workers deal with the clients? How so? Can you explain?

1. What do you think about the environment of the health facility?

- Is it comfortable and welcoming enough for women who visit for immunization?
- Are there an adequate number of health service providers?
- PROBE:
  - 1. External environment (building, garden, space)
    2. Physical facilities
    3. Sanitation and cleanliness
    4. Safety
    5. Materials and equipment
- In your opinion, how does the health facility’s atmosphere affect the experiences of the people seeking services from this health facility?
  - 1. PROBE: Demand for services?
    2. PROBE: Quality of services?
    3. How does it affect immunization, its uptake and continuation?
- What efforts has the Palika made over the years to improve the environment, if any?
  - 1. How effective were these efforts? Why?
    2. What could be done to make the health facility more welcoming for women and children to improve immunization rates?

1. Can you tell me about the health facility and Palika collaboration? How would you describe that relationship?

- Can you tell me if there have been any changes in the ways Palika and the health facility have collaborated in the past few years?
- Was there any budget allocated to the health facility after you were elected in Palika? If Yes, what were the budget for?
- Is there something related with improvement in facility environment? If Yes, was the project implemented? How were the effects?
- In your opinion, what needs to be done in the health facility to improve its environment? How could Palika collaborate in this?

**Intervention Feasibility**

1. We are planning on adjusting the clinic environment and facilitating provider-caregiver interactions in our research. We may paint walls, provide seating, and plant trees to make the clinic more visually appealing. We may also implement a new appointment system and encourage certain ways of speaking with caregivers to improve efficiency and communication. What do you think about these types of changes? What do you think caregivers will say to that?

*If the respondent finds the intervention problematic*

- Why do you think it may be a problem? Is there any way that we can make it more acceptable to you and other providers? To caregivers?

We have come to an end of this discussion. Do you have any questions for me?

Thank you very much for your time and patience.
