# Supplementary material for: The last stretch: Barriers to and facilitators of full immunization among children in Nepal’s Makwanpur District, results from a qualitative study
Source: PLoS One. 2022 Jan 21;17(1):e0261905. doi: 10.1371/journal.pone.0261905 (PMC8782481; doi:10.1371/journal.pone.0261905)
Supplement: S5 File — (DOCX) [file pone.0261905.s005.docx]

Cover Page (Interviewer’s Responses)

Rejoice Architecture Interview Guide

**Female Community Health Volunteers**

Interviewer’s name: _____________________

Interview number: _________

Palika: ____________________

Ward Number: _________

Village: ____________

Health facility Name: _____________

Respondent ID: __________

Thank you so much for taking time out of your day to participate in this interview.

Do you have any questions about the research and your participation before we begin?

**Warm-Up Questions**

1. Can you tell me about yourself? How long have you been working as FCHV? How many households and what area do you cover?
2. What are your key roles and responsibilities?

- PROBE: What are your responsibilities with respect to immunization, if any? Can you explain a bit?

**Immunization status**

1. Are you aware about the immunization status of your coverage area? If yes, can you tell me about it? How many of the children in your area would you say are up to date on their vaccines?
   - Are there children of vaccination age, who avoid/ miss out on vaccine dosages? What are their reasons for not vaccinating? Can you tell me about the reasons in brief?
2. I have noticed that some health facilities are clean, well-managed, and very efficient, whereas others are not so well run. Can you tell me about the health facilities in this area?
   - PROBE: For example, tell me a bit about whether they are crowded or not, whether they are clean or not, and how they function, overall
   - In your opinion, what could be done to make the health facilities more friendly toward people?

**Drivers & Barriers**

1. Can you tell me what activities/efforts/initiations that you or other FCHVs have made to improve vaccine coverage, if any?
   - How effective have these activities been?
   - What has worked? What might be the reasons?
   - What has not worked? What might be the reasons?
2. Are there any specific groups in the community with lower coverage?
   - If Yes, which are these groups?
   - Why do they have low coverage levels?
   - PROBE: Do they know about vaccines? What are their attitudes toward vaccines? Can they get to vaccination sites? Are vaccines too expensive? What else might be causing these groups to have low coverage?
3. What efforts have you or other FCHVs have made to improve vaccine coverage for these groups, if any?
   - - How effective have these activities been?
     - What has worked? What might be the reasons?
     - What has not worked? What might be the reasons?
4. We have learned that some women vaccinate their children all the way through 15 months, whereas others stop before all the vaccinations are done. Why do you think this is so?
   - PROBE: Why do you think some women stop vaccinating their children?
   - What could we do to ensure women come to their child’s next immunization visits? Do you have any experience from similar efforts?
5. How easy is it for the average parent to get their child vaccinated? Do you think mothers in this community have a hard time getting their children vaccinated? What do you think are some of their challenges?
   - Can women in this village overcome these challenges? What would help?

**Norms**

1. Please think about most women who live in this community. In your opinion, what do most women think about vaccines?
   - What is driving these perceptions?
   - Is there any pressure to get children vaccinated?
   - PROBE: How much pressure exists? Where does this pressure come from? (PROBE: family, friends, health systems, etc.)
   - What efforts have you or other FCHVs have made to counter negative perceptions toward vaccines in the community, if any?
     1. How effective have these activities been? How well liked were they?
     2. What has worked? What might be the reasons?
     3. What has not worked? What might be the reasons?
     4. Anything else?

**Communication**

1. What questions and concerns do you receive from the community regarding the immunization, if any? Can you share some of those?
   - How do you answer them?
   - What are the key messages that you transfer to the community?
   - Do you believe you have been trained adequately? How confident do you feel sharing your knowledge about immunizations with others?
   - Is there an area you would like to improve? Knowledge of vaccines? Key messages? Communicating with others?

**Health facility Atmosphere**

1. Can you tell me how you feel when you reach the health facility? (Pleasant, Energized, Unpleasant, Tired, Disgusting)

- What makes you feel that way?
- How does this affect the way you work and deal with the clients, if at all? Can you explain?

1. What do you think about the environment of the health facility?

- Is it comfortable and welcoming enough for women who visit for immunization?
- PROBE:
  - 1. External environment (building, garden, space)
    2. Physical facilities
    3. Sanitation and cleanliness
    4. Safety
    5. Materials and equipment
- In your opinion, how does the health facility’s atmosphere affect the experiences of the people seeking services from this health facility?
  - 1. PROBE: Demand for services? Quality of services?
    2. How does it affect immunization, its uptake and continuation?
- Have you noticed the facility’s environment changing over the years? Has it gotten better or worse?
  - 1. How does it compare with other health facilities in the area? Private clinics?
    2. What could be done to make the health facility more welcoming for women and children to improve immunization?

**Intervention Feasibility**

1. We are planning on adjusting the clinic environment and facilitating provider-caregiver interactions in our research. We may paint walls, provide seating, and plant trees to make the clinic more visually appealing. We may also implement a new appointment system and encourage certain ways of speaking with caregivers to improve efficiency and communication. What do you think about these types of changes? What do you think caregivers will say to that?

*If the respondent finds the intervention problematic*

- Why do you think it may be a problem? Is there any way that we can make it more acceptable to you and other providers? To caregivers?

We have come to an end of this discussion. Do you have any questions for me?

Thank you very much for your time and patience.
