# Supplementary material for: The last stretch: Barriers to and facilitators of full immunization among children in Nepal’s Makwanpur District, results from a qualitative study
Source: PLoS One. 2022 Jan 21;17(1):e0261905. doi: 10.1371/journal.pone.0261905 (PMC8782481; doi:10.1371/journal.pone.0261905)
Supplement: S6 File — (DOCX) [file pone.0261905.s006.docx]

Cover Page (Interviewer’s Responses)

Rejoice Architecture Interview Guide

**Father**

Interviewer’s name: _____________________

Interview number: _________

Palika: ____________________

Ward Number: _________

Village: _____________

Health Facility Name: _____________

Respondent ID: __________

Thank you so much for taking time out of your day to participate in this interview.

Do you have any questions about the research and your participation before we begin?

**Warm-Up Questions**

I’m new to (village name). Can you tell me a little bit about it? For example, what kind of crops do people grow here? What are the places to visit around? And you are a new dad, right? What is the name of your child? And how old is he/she? (MEMORIZE THE NAME, AS THAT WILL BE USED THROUGHOUT THE INTERVIEW)

*If child is older than 2 years, skip to end of interview*

**Vaccination Experience**

Now we will discuss your child’s vaccinations and your experiences regarding vaccination.

1. Has NAME been immunized?
2. (If yes) How did you hear about the immunization? Where did your child receive their vaccines– local health facility or somewhere else?
   - How was the immunization process organized? Was it an event or ongoing at the facility?
   - How many vaccines has NAME received? Do you know how many more vaccines NAME has to receive? Can you tell me when NAME has to receive them? *(If he does not know, ask him to guess)*
3. (If no) Why has NAME not been immunized?
   - PROBE: What is your opinion about NAME being immunized? Do you think it is good or bad for NAME?
   - Are you planning to get NAME immunized later?
4. What do you think about how well vaccines work?
   - PROBE: In your opinion, what do they do? Are there any benefits? What kind of benefits are there?
   - PROBE: Are there any negative sides to them? Can you tell me what they are?
   - PROBE: Who informed you about vaccines? How did they inform you?

**Involvement in Child Health**

1. How much input have you had in deciding whether or not NAME should be vaccinated? Why?
   - PROBE: How would you feel about having more input? Less input?
2. Who is typically the person to take NAME to his/her immunization visits?
   - PROBE: Why does _____ take NAME to their immunization visits?
   - What about other health visits for NAME? Who takes NAME?
   - PROBE: Why does ______ take NAME to his/her health visits?
3. If you do not do so already, how would you feel about taking NAME to one of his/her immunization visits?
   - PROBE: Why would you feel this way?
4. Have you and NAME’s mother had conversations about vaccines?
   - (If yes) What have you said during these conversations? What has she said?
   - (If no) Why not? What would you say to her if you did?

**Decision-Making**

1. Some people believe that the husband should decide how household wealth is spent. Others believe that the wife should help with that decision. Where does your opinion fall?
2. Who in your family makes the final decision about healthcare? Is it a joint decision?
3. What about deciding to visit family and friends? Who makes the final decision? Is it a joint decision?

**Child Aspirations**

1. How far do you want NAME to go in his/her schooling? Why?
   - PROBE: primary school? Secondary school? University?
2. After NAME finishes school, what sort of career do you want NAME to have?

**Vaccination Facilitators and Barriers**

1. When you think about immunization, what comes to your mind? What are the things that you think about? These could be positive things or challenging things. (improving health, side effects, health facility environment, cost)
2. Would it be difficult to get to (vaccination site)? What are the challenges?
3. Would it be difficult for you to get NAME vaccinated? What are the difficulties?

- PROBE: Anything else (that makes it difficult)?

1. We have learned that some parents vaccinate their children all the way through 15 months, whereas others stop before all the vaccinations are done. Why do you think this is so? Why do you think some parents stop vaccinating their children?

Thank you for answering all of my questions. Is there anything else you would like to add? Do you have any questions for me?
