# Supplementary material for: The last stretch: Barriers to and facilitators of full immunization among children in Nepal’s Makwanpur District, results from a qualitative study
Source: PLoS One. 2022 Jan 21;17(1):e0261905. doi: 10.1371/journal.pone.0261905 (PMC8782481; doi:10.1371/journal.pone.0261905)
Supplement: Attachment — Submitted filename: Response to reviewers.docx [file pone.0261905.s007.docx]

Response to Reviewers

We are grateful to the reviewers and editors for their comments. We have addressed all of them, as noted below.

**Response to Editor Comments:**

Please ensure that your manuscript meets PLOS ONE's style requirements, including those for file naming.

We have reviewed PLOS ONE’s style guidelines and have made the appropriate adjustments. If there are specific formatting requirements we have missed, please let us know and we will happily address them.

Title: Missing is the target group, i.e., 'in children'. Consider adding to the title.

We have revised the title to include the target group (children) and specify the district in Nepal where the study was conducted (Makwanpur).

Methods: Provide additional information on the characteristics of the geographical location of the study, such as population demographics and health burden, particularly in young children.

We have added information to the Study Setting section on Makwanpur’s population size, land area, geographic diversity, ethnic diversity, and child immunization rate. Other health indicators are often reported at the Regional level in Nepal, and so District-level data are not readily available.

Results: Why do some subheadings not include quotes from participants while most of the others do?

All subheadings now include quotes from participants.

Discussion: Strive to better organize as well as shorten its length.

The discussion section has been reorganized to follow the organization of the results section: demand-side factors, supply-side factors, and issues of trust at the intersection. Then, we have grouped our intervention implications into one sub-section labeled “Recommendations”. We have also cut down on the length by focusing only on the most prominent takeaways and conclusions. We hope this will help to flow more coherently for the reader.

**Response to Reviewer 1’s Comments:**

It would be appropriate to separately summarise the findings of quantitative studies conducted in Nepal related to the topic in the introduction section.

Due to the limited number of studies conducted among our target population in Nepal, specifically, we feel it is best to present the information together rather than separated out by study design.

The objective of this study is not clearly mentioned. A reasonable rationale for conducting this study is required.
To improve clarity of our study’s objectives, we have removed the sentences elaborating on our objectives and those of the umbrella study, the Rejoice Architecture Project. We hope by simplifying our objective statement and keeping it consistent with the abstract, the aims of the manuscript will be clearer. Additionally, we have stated that part of the objective of this study is to inform the intervention design for the Rejoice Architecture Project as justification for conducting the study.

Then coming to the result section, it is again not clear what are the themes actually about… themes are not reflective of results most often… the authors should not confuse the demand side with the quote from health workers… based on the title of this study, separate headings on facilitators and barriers and themes within those headings could be done.

The themes have been re-organized to align with the objective section. They are now broken down into 1. Demand-side facilitators & barriers, 2. Supply-side facilitators and barriers, and 3. Lack of mutual trust between the demand- and supply-sides.

Overall, there appeared to be a poorly consistent flow through the objectives, results, discussion, and conclusion. The discussion and conclusion section should be coherently organised as per the objective of the study and rewritten.

We have reorganized the results and discussion sections based on the reviewers’ feedback to follow the structure of our objectives section. We hope this resolves the issue of flow throughout the paper.

Also, the authors should mention why there are no quotes from fathers at all. Was the information not at all noteworthy? It should be explained in the manuscript.

Quotes from fathers largely reiterated the themes presented by the mothers/caregivers. We felt quotes from mothers would be stronger, as they were the target population on the demand-side. However, we have added a quote from a father under demand-side barriers.

**Introduction**

Line 59 – NIP, in 1977, was introduced as an immunisation program in the country, not to address inequities in vaccine coverage.

This statement has been removed.

Line 84 – reference number 13 is not appropriate to cite here as it was carried out in Kenya. Also, reference 12 focused on vaccines scheduled to be completed by 12 months of age. Would you please cite the appropriate reference here?

Our literature review is not limited to studies conducted in Nepal. Although we aim to highlight these studies as much as possible, we include references to studies conducted in other low- and middle-income countries which have similar barriers and facilitators to immunization as those in Nepal.

Lines 115 to 116: "In Nepal, health facilities are often crowded, have inadequate waiting spaces if any at all, and are often unclean." It is such a generalised sentence. It should either have a precise citation or should be removed.
This sentence has been removed.

**Methods**

Although a qualitative method is stated, no specific study design has been mentioned. Would you please keep the heading study design and mention it?

We have specified that we used a grounded theory design.

Lines 131 to 132 – Being accessible from Kathmandu doesn't make it suitable for the research. The other two reasons are quite fair.

We have revised this statement and elaborated on the selection of this District; namely, it’s representation of the larger country.

Line 135 – I wonder why individuals living within a 1 km radius of health facilities were only eligible participants as the distance to health facilities could be a barrier, especially in hilly regions of Nepal.
We have noted in the paper that the catchment areas of the health facilities are 1-km in radius. Therefore, sampling outside of this radius would have resulted in including participants who did not visit the study facilities.

Line 138 - Are there any reasons why mothers were not included in in-depth interviews?

In the Objectives section, we have clarified that this formative work is informing a social norms-based intervention focusing on mothers of young children and health workers. Therefore, a key domain of interest was norms related to immunization, which we convey through the descriptions of the instruments. We have clarified in the Participants section that for mothers, we were particularly interested in understanding the ways in which they communicate with each other about immunization.

Line 172 – Data collection procedure?? Also, please mention the data collection period as it could be valuable data.
The first part of the comment was not entirely clear to us (but we did add that data were collected “in-person.” And we have added the data collection period, as suggested.

Lines 190 to 192 – Could you please associate the reference cited with this study? If not, you need to mention the procedure here clearly.

This reference was cited earlier in the paper on page 6 under the Study Objectives section as the protocol for the Rejoice Architecture Project, the parent study of this research.

Lines 195 to 197– Providing enough context and clarity doesn't justify why member checking was not done to increase the credibility of the research.

This is a limitation of the study, and we have noted it in the Limitation section

Line 199 – Does it mean each interview and focus group discussion covered a separate topic? If so, it limits the transferability of the research. Can you please explain?

No. In the Instruments section, we describe that all instruments cover the same three topics (i.e., facilitators, barriers, and social norms), and that each instrument also covers other topics in addition to these three topics (e.g., the in-depth interview guide covers family experiences, the key informant guide covers local coverage, etc.).

Line 200 – The instruments should only be a guideline. It is not clear whether there was a separate guideline for each topic. Also, please include interview guidelines as an additional file.
Each topic had its own semi-structured questions with suggested probes. Data collectors were trained to follow the instruments as a guide, but let the conversation flow as needed, as we indicate on page 11 of the Instruments section.

The direct link to the registered interview and focus group instruments have been added to the references (ref no. 24). We have also uploaded the instruments as supplemental files.

**Results**

Line 307 - Here, the gender norm of women being the primary caretaker does not justify being a facilitating factor to increase vaccine coverage. Instead, the link between women receiving immunisation awareness when participating in mothers' groups and being the child's primary caretaker increased the likelihood of vaccinating their child.
This section was aiming to describe how gender norms influenced the immunization practices and experiences in the community, rather than the likelihood of getting vaccinated. We have removed it, as it was not a strong theme in regard to facilitators of immunization.

Line 344 – The last paragraph within this sub-theme is more of accountability issue and mistrust as represented by the theme, not a power imbalance.
We re-evaluated this paragraph and have moved it to the “Clients perceive micro-aggressions” sub-theme, as it depicts clients adapting their behavior (i.e., going to private facilities) to avoid feelings of discrimination and disrespect at public facilities.

Line 364 – Is 5-hours a regular office opening hours, or was it because of the absence of health workers in the facilities to provide service. Immunisation has been covered mainly as an outreach service in communities in Nepal. Does the above-mentioned service hour refer to it? Would you please address this clearly?

Health facilities offer many other services than immunization, and so their opening hours are not a reflection of immunization outreach. Health posts should be open for at least 6 hours per day from 10am-4pm. Although we cannot confirm the exact hours these facilities were open, we have edited this sentence to clarify that, in general, participants were unsatisfied with the few hours per day the health facilities were open for services.

Line 380 to 383 – It is not acceptable to include information from the researcher's perspective in the result section, especially in this type of study. If it is explained based on the participants' response, please remove "Generally speaking" and support this claim with participants' quote.

This paragraph is not from the researcher’s perspective, but a summary of the general findings from health worker reports. We have revised this paragraph to make this clear.

Line 384 to 389 – This is going out of context unless linked with the study's objective. Are they talking about addressing adverse effects of immunisation or the mistrust due to previous experiences on immunising their child? Or are they just expressing their other concerns related to health care services?

We have removed this paragraph as it did not align well with the sub-heading and was not a strong enough or relevant enough finding to pull out on its own.

Line 394 – This sentence does not reflect the essence of the quote here. It is more about not getting other services if not fully vaccinate their child. Refusing to immunise a child by health worker is a sensitive claim, and therefore a quote is required.

We have revised this sentence to more accurately set up the following quotes.

Line 431 – Why family planning devices is affecting immunisation efforts?
Family planning devices are just one example of how a lack of resources is negatively affecting service delivery. To avoid confusion, we have removed this example.

Line 445 to 453 – Why the results are not linked with immunisation here? It looks more like an interview related to the condition of the health care facility rather than how the health facility environment affects vaccine uptake.

The health facility environment affects not only immunization service delivery, but delivery of all services offered in those facilities. We have added a clause to make this salient.

Line 454 – Provider or Provider’s

**Discussion**

Line 518 – Time hasn't been explained clearly as a barrier in the result section in the way it is mentioned here in the discussion.
This section has been removed.

Line 524 to 526 – It is expressed from the key informants on the supply side rather than the family caregiver in the result section. It is misleading.

This section has been removed.

Overall, the discussion should be coherently organised as per the objective of the study and rewritten.
Please see our response above.

**Limitations**

Line 588 to 589 – "It is also a fertile district able to grow and export cash crops to neighbouring cities of 589 Kathmandu and Hetauda" – This sentence has nothing to do with this study unless the socio-economic status of study participants is linked with vaccine uptake.

This sentence has been removed.

There could be more limitation
– one could be member checking (as mentioned earlier)

This has been noted
- Including participants within 1 km radius excluded participants who could be affected by travel time to health facilities

This has been noted
- No information is provided about the participants' backgrounds. It would be worthy of increasing the transferability of the study findings.

This is a tough one. We believe the most relevant background is that Makwanpur is a district with much greater access to urban facilities (in Kathmandu and Hetauda) than other districts in Nepal. This reduces generalizability, but the title now contains the name of the district, which communicates where the study was done (and hence the extent of appropriate generalizability).
- Was the number of participants finalised beforehand? Or was it based on data saturation?
This was done beforehand, because our IRB requires us to provide information about the sample size.

**Conclusion**

Line 594 – "stocking adequate doses of vaccines" - not mentioned in the result section and, therefore, cannot be included in the conclusion

Line 595 – "people's perceptions that many others do not fully vaccinate their children" – How is this a barrier? And why is it on conclusion rather than other important barriers discussed in the result section?

Line 596 – "feelings of experienced disrespect" – What does this mean?
We have revised our examples here to reflect the key themes from the results section.

Line 608 – There's nowhere in the results section that travel time is a barrier.

We have removed this barrier, as we do not delve into this issue in this paper.

Line 617 – Who are these marginalised groups? There is no identification of marginalised groups' deviant behaviours in the results.
We have revised this to reference clients more generally.

Overall, the conclusion should be rewritten.

**Response to Reviewer 2’s Comments:**

The sample size is somewhat an issue, given the information that the work is trying to capture.

We assume that the reviewer is noting that the sample size was not large enough. This is difficult to ascertain, of course, but we did find repetitions of themes in the data, suggesting that we were close to saturation. We should also note that the title of the paper now has the name of the district, which communicates that findings pertain to the one district.

The one district (out of 77 in the country) that was chosen is in close proximity to the capital of Nepal, Kathmandu and as such it may not truly represent the situation prevalent in rest of Nepal. The authors should make sure that this is identified as a major limitation of the study.

We have elaborated on the representativeness of Makwanapur in the Study Setting section.

The current title of the study and its objectives do not match the results provided. Perhaps the title and objectives of the study should be revised to truly reflect the work carried out.

The title and objectives are addressed above.
