# Supplementary material for: The last stretch: Barriers to and facilitators of full immunization among children in Nepal’s Makwanpur District, results from a qualitative study
Source: PLoS One. 2022 Jan 21;17(1):e0261905. doi: 10.1371/journal.pone.0261905 (PMC8782481; doi:10.1371/journal.pone.0261905)
Supplement: Attachment — Submitted filename: Response to Reviewers.docx [file pone.0261905.s008.docx]

PONE-D-21-16867R1

The last stretch: Barriers to and facilitators of full immunization among children in Nepal’s Makwanpur District, results from a qualitative study

**Response to Reviewers**

We are grateful to the reviewers and editors for their comments. We have addressed all of them, as noted below.

**Journal Requirements:** Please review your reference list to ensure that it is complete and correct. If you have cited papers that have been retracted, please include the rationale for doing so in the manuscript text, or remove these references and replace them with relevant current references. Any changes to the reference list should be mentioned in the rebuttal letter that accompanies your revised manuscript. If you need to cite a retracted article, indicate the article’s retracted status in the References list and also include a citation and full reference for the retraction notice.

In this revision, we have made no changes to our reference list. No references have been retracted. We confirm that it is complete and correct.

**Comments to the Author**

6. Review Comments to the Author

Reviewer #1: I highly appreciate the authors’ for thoroughly revisiting the manuscript as required. I have very few minor disagreements.
1. Line 84
I still disagree that this sentence—Nepal requires seven separate visits over 15 months to complete the full schedule [12,13]—is a generalised sentence that represents results from other low- and middle-income countries as it specifically mentions about Nepal. Therefore, I would suggest to either remove the citation from Kenya or make the sentence more generalised.

Thank you for pointing this out. We agree. We have removed this reference from line 84.

2. Line 178
The title of this section is only ‘Procedure’. As it is the data collection procedure, it would be better to have the clear title for this section.

Thank you for clarifying this comment. We have updated the title of this section to be “Data Collection Procedure”.

3. The same information is repeated in line 254 (generational shift in attitudes toward immunization) and line 256. Please avoid repeating the same information, especially within the same section.

Thank you for pointing this out. We have removed the sentence which began on line 256.
